# Visually evoked potentials (VEPs) across the visual field in hearing and deaf cats

**DOI:** 10.3389/fnins.2023.997357

**Published:** 2023-03-03

**Authors:** Thomas Mitzelfelt, Xiaohan Bao, Paisley Barnes, Stephen G. Lomber

**Affiliations:** ^1^Department of Physiology, McGill University, Montreal, QC, Canada; ^2^Integrated Program in Neuroscience, McGill University, Montreal, QC, Canada

**Keywords:** deafness, hearing loss, cross-modal plasticity, cortical magnification, EEG

## Abstract

**Introduction:**

Congenitally deaf cats perform better on visual localization tasks than hearing cats, and this advantage has been attributed to the posterior auditory field. Successful visual localization requires both visual processing of the target and timely generation of an action to approach the target. Activation of auditory cortex in deaf subjects during visual localization in the peripheral visual field can occur either *via* bottom-up stimulus-driven and/or top-down goal-directed pathways.

**Methods:**

In this study, we recorded visually evoked potentials (VEPs) in response to a reversing checkerboard stimulus presented in the hemifield contralateral to the recorded hemisphere in both hearing and deaf cats under light anesthesia.

**Results:**

Although VEP amplitudes and latencies were systematically modulated by stimulus eccentricity, we found little evidence of changes in VEP in deaf cats that can explain their behavioral advantage. A statistical trend was observed, showing larger peak amplitudes and shorter peak latencies in deaf subjects for stimuli in the near- and mid-peripheral field. Additionally, latency of the P1 wave component had a larger inter-sweep variation in deaf subjects.

**Discussion:**

Our results suggested that cross-modal plasticity following deafness does not play a major part in cortical processing of the peripheral visual field when the “vision for action” system is not recruited.

## Introduction

Visual enhancement in the far-peripheral visual field of congenitally deaf humans and animals is one of the most impressive examples of compensatory cross-modal plasticity (Neville and Lawson, 1987; Bavelier et al., 2000; Lomber et al., 2010). In cats, the neural mechanism of this observed enhancement has been demonstrated to involve deaf auditory cortex. Deactivation of the region posterior to primary auditory cortex (i.e., the posterior auditory field, PAF) decreases enhanced visual localization performance in deaf cats to a level no different from hearing cats, indicating its involvement (Lomber et al., 2010). It is particularly interesting that the greatest enhancement of visual localization in deaf cats is observed at the largest eccentricities (furthest into the periphery), suggesting they rely more on visual cues for orientation behavior. Conversely, in hearing cats, auditory cues contribute more significantly to orientation behavior whereas visual cues in the far periphery are less significant.

Visual orientation, in its most general definition, includes any visually guided behavior where gaze is redirected toward a target, e.g., predator or prey (primates: Lisberger and Fuchs, 1978a,b; grasshopper: Szentesi et al., 1996; cat: Lomber and Payne, 2004), through movement in the body, head, and/or eyes (gaze). Orientation behavior is a vital neurological function for survival that involves both cortical and subcortical circuits. The superior colliculus (SC) is deemed as the most prominent modality-unspecific sensorimotor hub in mammals (King, 2004), which acquires the retinotopic map of its direct visual inputs from retinal ganglion neurons and receives modulation of its indirect inputs from cerebral cortex (Schiller et al., 1974; Mize and Murphy, 1976). In primate cortex, it has been identified that neurons in the frontal eye field respond specifically to locations in the visual field, that correspond to either the location of the visual stimulus or the destination of the intended saccade (Thompson et al., 2005). It is proposed that visual orientation (or saccadic eye movement, to be more specific) is triggered by a combination of neuronal activity related to both stimulus-driven bottom-up visual processing and goal-directed top-down motor generation. Human behavioral experiments have shown that subjects can make faster saccade responses when the visual stimulus is more salient, or when the target location is easier to predict (Marino and Munoz, 2009).

Considering permanent lesion or reversible deactivation studies, cat area 5 and part of area 6 have been found to be essential for the action of orientation behavior, as their deactivation eliminates both acoustic and visual localization (Malhotra et al., 2004). Some other areas are essential for acoustic-specific orientation, such as the primary auditory cortex, PAF, and anterior ectosylvian sulcus (AES) (Malhotra et al., 2004). Areas such as the posterior middle suprasylvian sulcus (pMS), dorsal posterior ectosylvian gyrus (dPE), and posterior suprasylvian sulcus (PS) are critical for visual orientation (Lomber and Payne, 2004). In cat auditory cortex, many areas including PAF contain neurons that respond to sound for selective source locations (Stecker et al., 2003; Lee and Middlebrooks, 2013). It has also been shown that AES in deaf cats is implicated in visual rather than auditory localization following cross-modal plastic changes (Meredith et al., 2011). It is unclear whether activity in AES contributes to the bottom-up, stimulus-driven, or top-down, goal-directed pathway.

Previous human studies have found that visually evoked potentials (VEPs) in congenital deaf subjects demonstrated shorter peak latency in early VEP component N85 (Hauthal et al., 2014), and larger magnitudes in P100 (Hauthal et al., 2014), N150, and P230 (Neville et al., 1983) when compared to normal hearing subjects. In cochlear-implanted children, shorter latency of the visual N1 component (Corina et al., 2017) and the higher occurrence and amplitude of oscillations in the N1-P2 complex (Campbell and Sharma, 2016) were found in comparison to normal hearing subjects. Brain imaging studies have also shown that areas in auditory cortex were activated by visual stimulus in deaf sign language users (Fine et al., 2005), in congenital deaf (Finney et al., 2001, 2003; Scott et al., 2014), when compared to hearing volunteers.

To investigate the stimulus-driven neural activity associated with visual localization, we recorded VEPs in response to a checkerboard stimulus present at one of seven eccentricity markers, between 0 and 90 degrees away from the midline in hearing and deaf cats under light anesthesia. Although VEPs are widely used in research and clinical applications (e.g., Laron et al., 2009), we were surprised to find that no existing documentation of VEP studies included far-peripheral stimuli as done previously in a cat visual localization task (Lomber et al., 2010). As expected, VEPs decayed exponentially with increasing eccentricity in both the hearing and deaf groups. However, we did not see a dramatic difference in VEPs between the two groups, suggesting a lack of cortical cross-modal plasticity involvement in sensory processing of the peripheral visual field.

## Materials and methods

All procedures were conducted in compliance with the National Research Council’s Guide for the Care and Use of Laboratory Animals (8th edition; 2011) and the Canadian Council on Animal Care’s Guide to the Care and Use of Experimental Animals (1993). Furthermore, the following procedures were approved by the Animal Care Committee for Faculty of Medicine and Health Sciences at McGill University.

### Deafening

Early deafness was produced in five cats using systemic ototoxic procedures. Before 21 days postnatal, three kittens received co-administration of subcutaneous kanamycin (300 mg/kg) and intravenous furosemide (2 mg/mL to effect; Valent Pharmaceuticals, Laval, QC, Canada). This drug combination has been identified to damage cochlear hair cells and induce profound, bilateral deafness (Schwaber et al., 1993). Throughout the procedure, auditory brainstem responses (ABRs) were measured to monitor the degree of hearing loss. Two other newborn cats received neomycin daily from the day of birth to between postnatal days 26 and 28 (Leake et al., 1991, 1997) before ABRs showed profound hearing loss. All five cats were confirmed deaf (click ABR threshold higher than 80 dB HL) in a follow-up ABR procedure at least 3 months later.

### Animal preparation and anesthesia

In total, 12 cats (7 hearing and 5 early deaf) were examined. One hearing subject was excluded for poor signal quality leaving both groups with comparable sex and age distribution (hearing: 71.4% female and mean age 4.2 years; deaf: 60% female and mean age 3.1 years). After the subject was sedated using 0.04 mg/kg dexmedetomidine (Dexdomitor, Zoetis) injected intramuscularly, the left eye was occluded using a hard black contact lens so that visual stimuli were presented unilaterally. Phenylephrine (Mydfrin, Alcon) was applied to the right eye to dilate the pupil, and saline drops were used as lubrication. Hearing subjects were also ear-plugged to minimize auditory input. During stimulus presentation and EEG recording, the anesthesia level was closely monitored and remained stable so that there were rarely any cases of artifact from subject movement in the raw signal. Data collection was terminated either by 45 min after the injection or at the end of the session during which any sign of subject movement was noticed. After removing the electrodes, contact lens, and ear plugs, the subject received an intramuscularly injection with 0.4 mg/kg atipamezole (Antisedan, Zoetis) to facilitate recovery from sedation. Given the large range of stimulus eccentricities selected (0–90 degree), the minor horizontal eye movements previously reported in anesthetized but non-paralyzed cats (O’Keefe and Berkley, 1991) should not confound the effect of stimulus eccentricity. As such, our subjects were maintained in centrally-gazed position without the use of neuromuscular blockers.

### Visual stimulation

**FIGURE 1:**
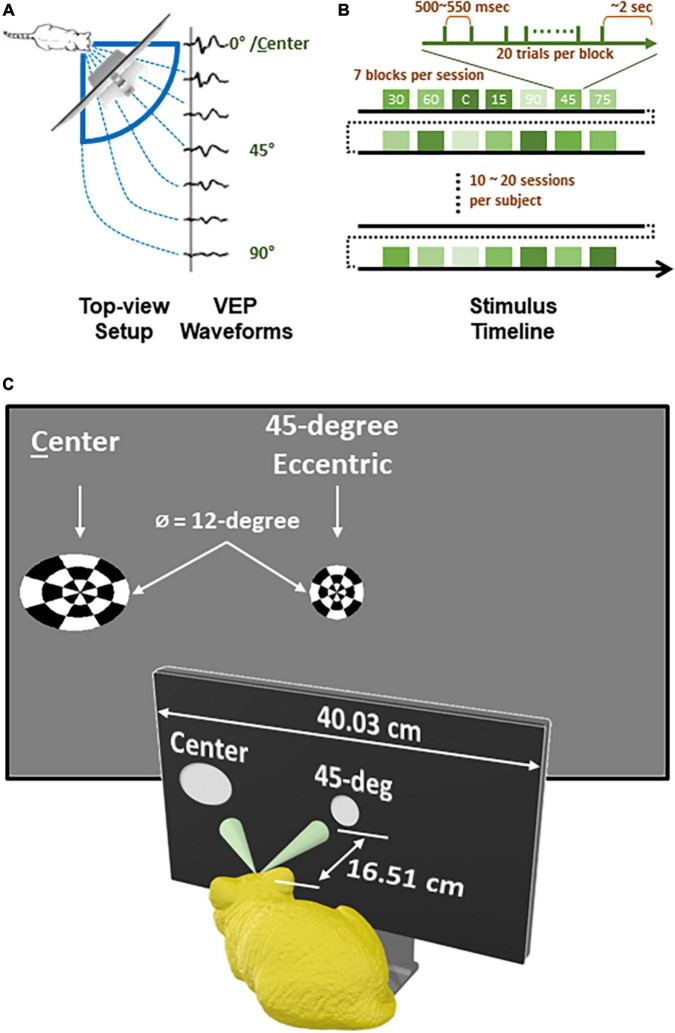
Experiment design and recording timeline of each subject. **(A)** A top-view diagram showing how checkerboard stimuli of varying eccentricity were present to animal subject and corresponding VEP waveforms for each eccentricity. **(B)** A timeline of recording for each subject. **(C)** Stimuli viewed from subject’s perspective.

The visual stimuli were presented to subjects from a 30-inch 2,560-by-1,600 LED screen with 178-degree horizontal and vertical viewing angles (Dell, U3014). To cover a wide azimuth range of visual field, the screen was placed 6.5 inch away from the right eye to the 45-deg front-right of subject (Figure 1A). The stimulus used to evoke VEPs was 12-deg-wide circular checkerboards with dartboard pattern (3 concentric rings, 8 divisions per ring). To occupy roughly the same size of visual field from the cat’s perspective, stimuli presented on the screen were the warped projection of the checkerboard, depending on how much stimulus eccentricity (0-, 15-, 30-, or 45-deg) deviated from 45 degrees (Figure 1C). For the same reason, the luminance of the bright cells in the checkerboard was calibrated to 100 cd/m^2^ individually for each eccentricity from different angles (Konica Minolta, LS160).

All stimuli were programmed and controlled with PsychToolbox (Brainard, 1997; Pelli, 1997; Kleiner et al., 2007) run on MATLAB, rendered by a graphic card (AMD, Radeon HD 6800 Series), and transited at a video processing unit (Cambridge Research System, Bits#) for generating stimulus timestamps. Once a subject was under stable sedation, EEG data was collected while visual stimuli were presented for 10 to 20 sessions (Figure 1B). Each session lasted for ∼1.5 min, consisting of 7 blocks for different eccentricities in randomized order. In each block, the stimulus inverted 20 times (i.e., 20 sweeps), with inter-sweep interval randomly set between 500 and 550 milliseconds. Between two blocks, there was a 2-second-long black screen. At the end of each session, the level of sedation was re-evaluated, with measurement of heart rate, SpO2, respiration, and electrode impedances taken if needed, before starting the next session.

### EEG recording and signal processing

Three 25G stainless steel needles were placed subcutaneously as recording electrodes. The active electrode was placed near the midpoint of subject’s interaural line, while the reference electrode was placed inferior to the right ear (ipsilateral to the side of visual stimulation). The ground electrode was placed on subject’s dorsum. The impedance of both active and reference electrodes was maintained below 3 kOhm during recording. The signal was amplified and digitized with a pre-amplifier (TDT, RA4LI/RA4PA), streamed onto a digital signal processor (TDT, RZ2), and stored on a computer hard drive.

All data analysis was performed offline. The signal was digitally filtered between 1 and 30 Hz (about 10 dB/octave roll-off) and notch-filtered at 30, 60, 120, 180, and 240 Hz, before epochs were extracted from 80-ms pre-stimulus to 400-ms post-stimulus. All epochs were demeaned with their pre-stimulus baselines individually, before being grouped by stimulus eccentricity and further processed separately (e.g., averaging or resampling).

### Data analysis

Root-mean-square values were obtained using MATLAB built-in function *rms()*. Each averaged waveform was separated into an 80-ms pre-stimulus zero-mean baseline window and 400-ms post-stimulus response window, producing two RMS values, respectively. The corrected RMS value was calculated as in the following equation:


$$RMS_{\text{corrected}}=\sqrt{RMS_{response}{}^{2}-RMS_{baseline}{}^{2}}$$

Curve fitting of the corrected RMS as a function of stimulus eccentricity was performed in MATLAB and its Curve Fitting Toolbox™. We first obtained the initial values for the model coefficients using function *polyfit()*, where the corrected RMS values were first converted into logarithmic space for exponential modeling. Then, function *fit()* was used to fine tune the model coefficients with customized anonymous functions *RMS*_*corrected*_ = *a*⋅*e^b^*^⋅*Eccentricity*^ for exponential modeling, and *RMS*_*corrected*_ = *k*⋅*Eccentricty* + *b* for linear modeling, and to evaluate the goodness of fit.

To analyze peak components (N1, P1, and N2 components), we first manually determined a window surrounding the candidate of interest by interactively overlaying a pair of cursors with the averaged VEP waveform. This process was visually guided with graphical information including polarity, amplitude, latency, as well as the entire waveform and other peak components. Bounded by this window, either a minimum or maximum was identified with MATLAB built-in function *min()* or *max()* for the calculation of amplitude and latency.

To quantify the inter-sweep variability, we chose 18 out of total 20 sweeps (90%) before averaging to generate 190 resampled waveforms for each stimulus eccentricity in each subject. Here we only included the first 10 sessions, even when there were more sessions recorded in some subjects. The reference latency was determined from the waveform averaged from all 200 sweeps in the same way as described above. In each resampled waveform, the peak with a latency closest to the reference latency was taken as a resampled latency. Probability density functions, which were re-centered to the reference latency, were given by MATLAB built-in function *ksdensity()*. Mean absolute deviations (MADs) were calculated using MATLAB built-in function *mad()*.

### Statistical analysis

For corrected RMS values, peak amplitudes and latencies of the three components, we adopted two-way mixed-design ANOVA with deafness as the between-group variable and stimulus eccentricity as the within-group variable. As the motivation of the current study is to compare VEP traces between hearing and deaf cats, we enforced the “simple effect” test at each of seven stimulus eccentricity levels, regardless of whether the main effect of deafness is significant. Due to a small sample size in this study, both parametric (two-sample Student’s *t*-test, *t-test2()* in MATLAB) and non-parametric (Mann–Whitney U test, *ranksum()* in MATLAB) methods were used, and the largest *p*-values of the two tests were used to report statistical significance, without correction for multiple comparison. For latency jitter MADs, data of different stimulus eccentricity from different subjects were pooled together for comparison between hearing and deaf cats. The effect of deafness on MADs was examined by ANCOVA with peak amplitude as a covariate. All statistics were performed in MATLAB and its Statistics and Machine Learning Toolbox™.

## Results

In this study, we recorded VEPs in response to checkerboards presented at seven different eccentricities, from 0 to 90 degrees, in seven hearing and five early deaf cats. The recorded data in all except for one hearing subject were of good quality, in respect to the signal-to-noise ratio, and were further analyzed and reported below.

### Waveforms

**FIGURE 2:**
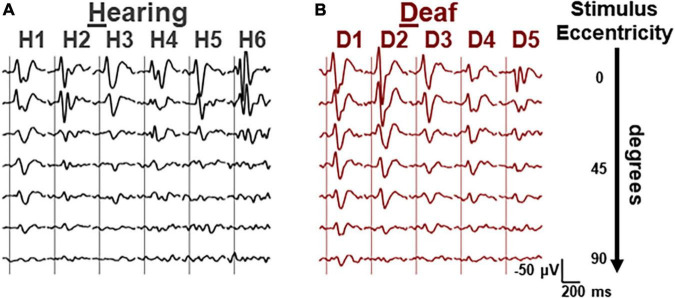
Averaged VEP waveforms for different eccentricities of each subject. **(A)** Waveforms from the data collected from 6 hearing cats, grouped by subject and stacked in each column. Stimulus eccentricity increases for the waveforms from the top (central) to the bottom (90 deg). **(B)** Waveforms of 6 deaf cats. Same scalars are applied to both hearing and deaf waveforms. Horizontal, 200 ms. Vertical, 50 μV, negative-up.

First, we vertically stacked the averaged VEP waveforms for each hearing and deaf subject in the order of stimulus eccentricity (Figure 2). For the smaller eccentricities (upper rows), the VEP waveforms were not distinctively different between the two groups. However, the VEP responses seemed to scale down more drastically in deaf subjects than in hearing subjects as stimulus eccentricity increased.

### Root-mean-square (RMS) level

**FIGURE 3:**
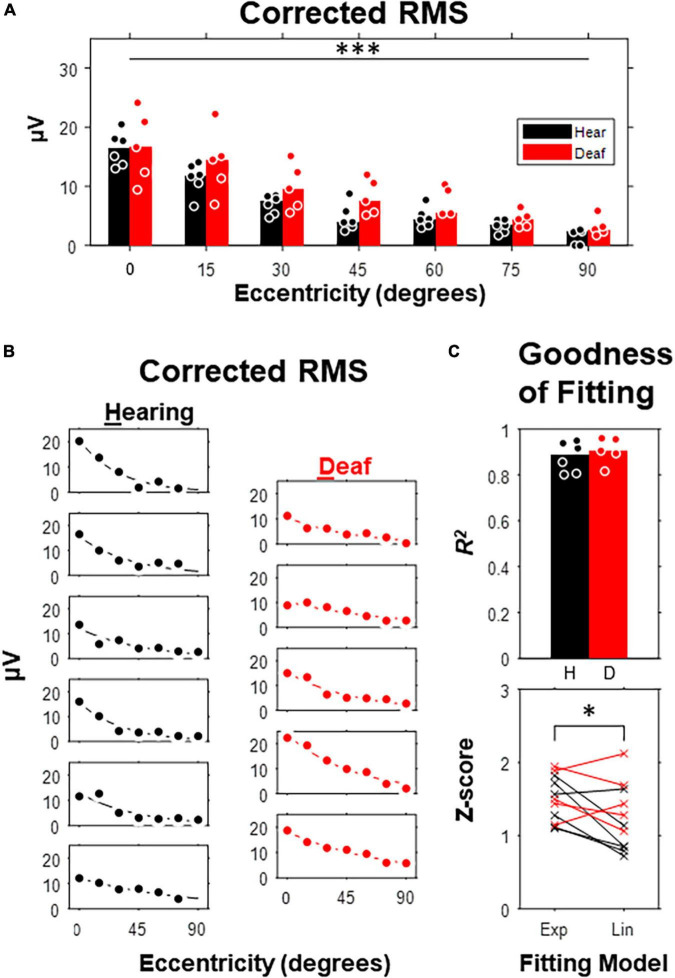
Corrected Root-Mean-Square (RMS) as a function of stimulus eccentricity. **(A)** RMS over a 420-ms post-stimulus window corrected with an 80-ms pre-stimulus baseline. Dot, data for individual subject. Bar, median. **(B)** Curve fitting with an exponentially decay model carried out for each of 6 hearing and 5 deaf subjects. **(C)** Goodness of fit. Top, adjusted *R*^2^ compared between hearing and deaf group. Bottom, Fisher’s Z scores of the adjusted *R*^2^ derived from exponential and linear fitting for each subject. *p < 0.05; ^***^p < 0.001 for the main effect of eccentricity.

**FIGURE 4:**
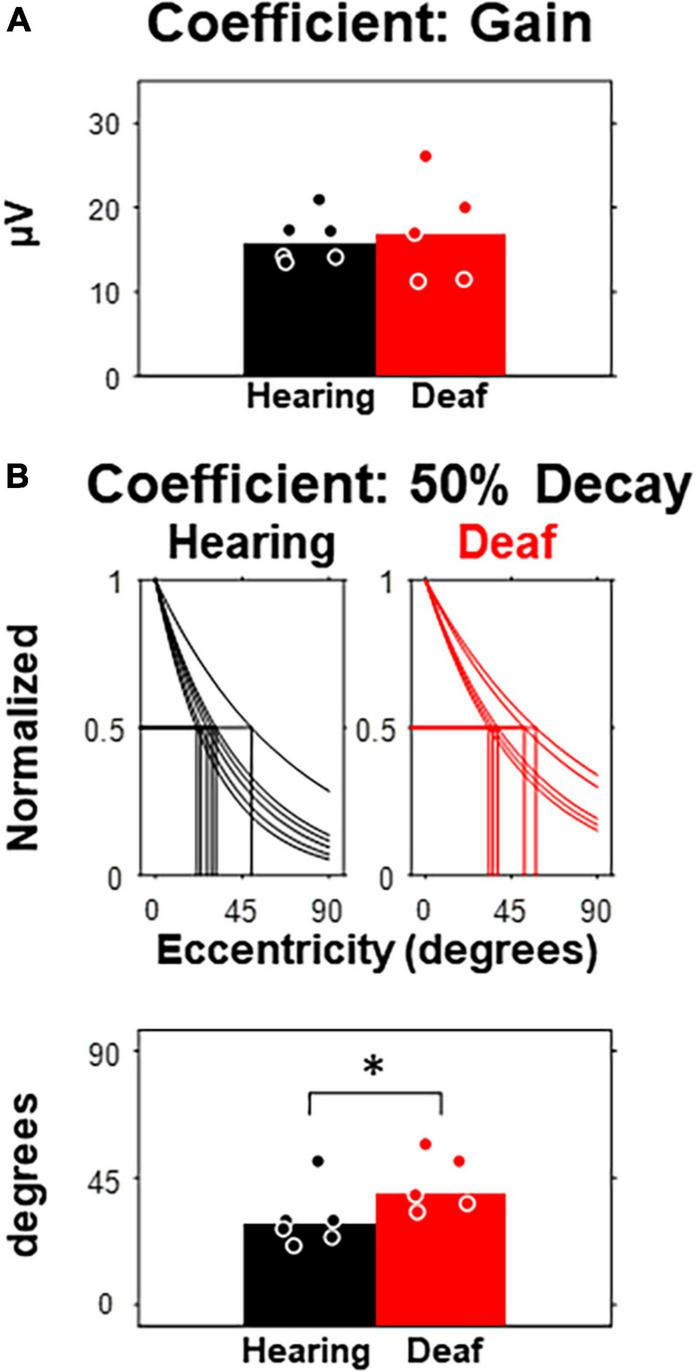
Comparison of model parameters between hearing and deaf subjects. **(A)** The fitting coefficient representing the gain of the model, which is equivalent to the model-estimated RMS at central eccentricity. **(B)** The fitting coefficient representing the rate of decay, which is equivalent to the amount of eccentricity increase that predicts 50% of RMS at central eccentricity. Top, model and 50% thresholds for each subject separated by group. Bottom, 50% threshold compared between hearing and deaf. *p < 0.05.

To quantify VEP responses with the least *in prior* bias, we calculated the noise-corrected root-mean-square (RMS) of the waveform in the entire post-stimulus window (i.e., 400-ms) for each subject and compared between hearing and deaf groups (Figure 3). In both groups, the median of the corrected RMSs decayed monotonously with increasing eccentricity (Figure 3A). A two-way mixed-design ANOVA showed that the effect of stimulus eccentricity was significant [*F*(6,54) = 52.02, *p* < 0.001], but not the effect of deafness or the interaction between eccentricity and deafness. For stimuli at the 45-degree and 60-degree eccentricities, the median of RMSs in the deaf group was higher than the hearing group with a large effect size quantified as Cohen’s *d* (see Table 1). We also investigated the effect of stimulus eccentricity and the trend of decay in RMS at the individual subject level (Figure 3B). Both non-linear and linear models were adopted to fit the RMSs as a function of eccentricity. All 11 subjects fit well with the two-coefficient (gain and decay) exponential model with an adjusted *r*^2^ above 0.75 (Figure 3C). Three deaf and five hearing subjects fit better with a non-linear exponential model than the linear model. A paired-test comparison of the Fisher’s Z-transformed adjusted *r*^2^ values showed that the advantage of the non-linear model was statistically significant (*t*_10_ = 2.26, *p* = 0.047 < 0.05). However, the gain yielded from the model fitting was not different between the two groups (Figure 4A). The decay rate was slightly higher in the deaf group (*U* = 42, *p* = 0.030 < 0.05) (Figure 4B).

**TABLE 1:** Effect size for the comparisons between the two groups.

	RMS	Peak amplitude			Peak latency		
		**N1**	**P1**	**N2**	**N1**	**P1**	**N2**
0	**−0.07**	**−0.28**	**−0.25**	**−0.12**	**−0.81**	**−0.38**	**−0.07**
15	**−0.57**	**−0.45**	**−0.53**	**−0.73**	**−0.2**	**−0.68**	0.05
30	**−0.93**	−1.77	−1.25	**−0.75**	**−0.13**	**−0.3**	**1.09**
45	−1.21	**−0.53**	−1.51	−1.14	0.77	0.74	**2.43**
60	−1.1	**−0.9**	−1.24	**−0.62**	0.06	0.02	0.52
75	**−0.86**	**−0.55**	**−0.94**	**−0.66**	0.34	0.25	0.88
90	**−0.54**	**−0.36**	**−0.59**	0.02	0.62	**−0.12**	0.01

Bold values represent the effect size larger than 1.

From the analysis of RMSs, we found that there was no main effect of early deafness on the overall amount of cortical activation by the checkerboard stimuli. The visual activation, however, was modulated by stimulus eccentricity exponentially, in an analogous way for both hearing and early deaf cats.

### N1-P1-N2 complex

Next, we investigated the VEPs by different peak components. The VEP waveforms of all subjects showed at least 3 components, which are referred as N1-P1-N2 complex and characterized by the pattern of their peak polarities, relative amplitudes, and peak latencies. According to the VEPs from the total 12 subjects in the current study, it started with a negative component N1 peaking at 54∼144 ms, followed by a positive component P1 peaking at 86 ∼ 189 ms, and ends with a second negative component N2 peaking at 178∼367 ms. In four subjects (3 hearing and 1 deaf), there were extra peaks between N1 and P1, which were not further analyzed.

**FIGURE 5:**
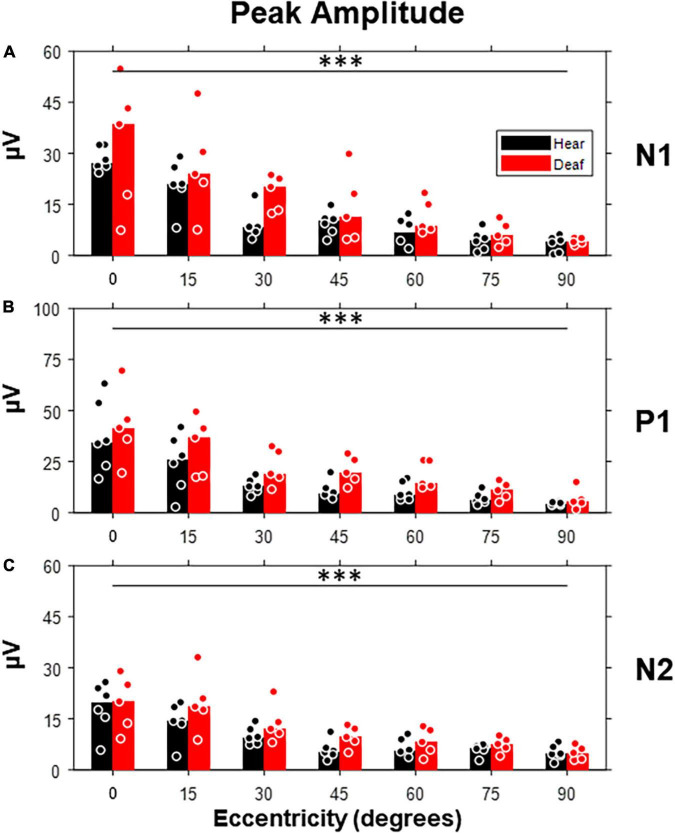
Peak amplitudes of three VEP components (N1, P1, and N2) as a function of stimulus eccentricity. **(A)** Peak amplitudes quantified from the N1 component, which is defined as the first negative deflection after stimulus onset. **(B)** Peak amplitudes quantified from the P1 component, which is defined as the first positive deflection after N1. **(C)** Peak amplitudes quantified from the N2 component, which is defined as the last negative deflection. Dot, individual subject; bar, median; ^***^p < 0.001 for the main effect of eccentricity.

We started with the exploration on how the peak amplitude of each component was affected by the stimulus eccentricity and early deafness (Figure 5). All three components showed a clear trend of decrease in amplitude with increasing eccentricity [N1: *F*(6,54) = 27.07, *p* < 0.001; P1: *F*(6,54) = 21.73, *p* < 0.001; N2: *F*(6,54) = 20.98, *p* < 0.001]. However, the difference between the hearing and deaf groups was not significant. For individual stimulus eccentricity, the deaf group showed larger peak amplitude with a large effect size (a) in the N1 component for stimuli present at 30-degree eccentricity, (b) in the P1 component for stimuli present at 30-, 45-, and 60-degree eccentricity, and (c) in the N2 component for stimuli present at 45-degree eccentricity, when compared to the hearing group (see Table 1).

**FIGURE 6:**
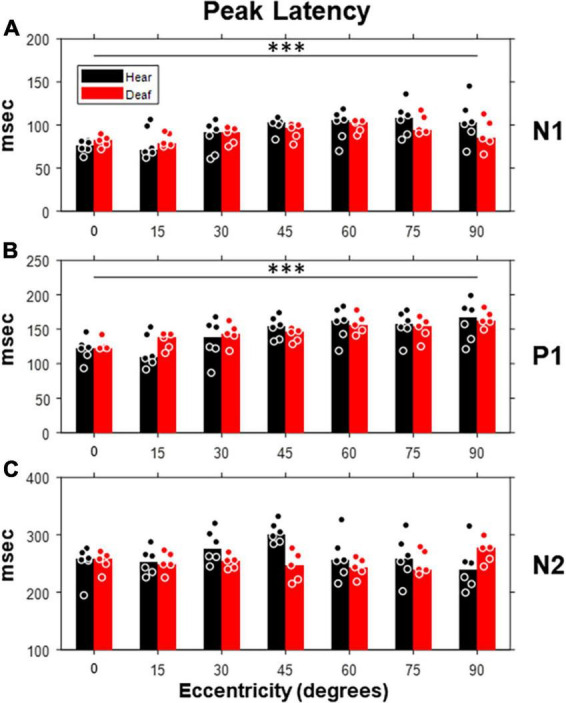
Peak latencies of three VEP components (N1, P1, and N2) as a function of stimulus eccentricity. Same layout of panels **(A–C)** as in Figure 5 but for peak latency. Dot, individual subject; bar, median; ***p < 0.001 for main effect of eccentricity.

We also examined how the peak latency of each component was affected by the stimulus eccentricity and deafness (Figure 6). Both N1 and P1, but not N2, showed prolonged latency with increasing eccentricity [N1: *F*(6,54) = 5.38, *p* < 0.001; P1: *F*(6,54) = 12.36, *p* < 0.001]. For individual stimulus eccentricity, only N2 component revealed a shorter peak latency in the deaf group than the hearing group [*F*(1,9) = 7.49, *p* = 0.023 < 0.05], especially for stimuli present at 30- and 45-degree eccentricity (see Table 1).

Although the increase of peak amplitude and shorter peak latency in early deaf cats reported above showed minimal statistical significance, they seemed to preferably occur for middle-peripheral visual field (e.g., 30-, and 45-degree eccentricities). In contrast to the lack of group differences found with RMS level, the trend of an increase in peak amplitude and a shorter peak latency suggests that the signal morphology was more sensitive to the effect of early deafness than the RMS values of VEPs. The trend we found is also consistent with previous studies of VEPs in deaf human subjects (Neville et al., 1983).

### Inter-sweep variability

**FIGURE 7:**
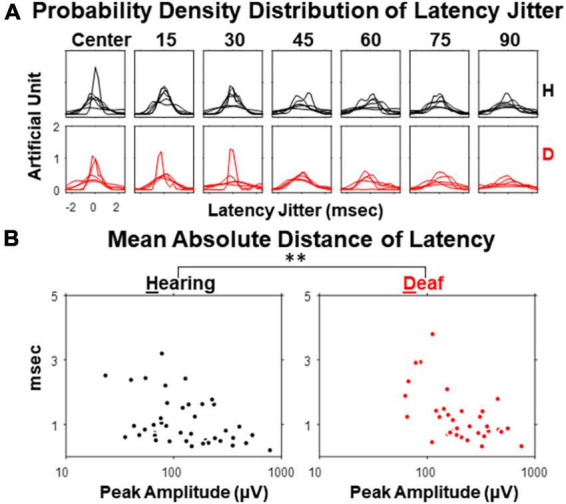
Jitter of peak latency for VEP P1 component in hearing and early deaf cats. **(A)** The estimated probability density for each of 7 stimulus eccentricities. Each line represents one of the subjects in both groups. **(B)** Mean absolute deviation (MAD) of resampled latency plot against the peak amplitude of the averaged waveform. Left, hearing. Right, deaf. Each dot represents one stimulus eccentricity in one subject. ^**^p < 0.01 for the main effect of deafness in ANCOVA.

Finally, we explored the inter-sweep variability of the peak latencies of N1 and P1 components. For each subject, 190 re-samples were taken by averaging 90% of the sweeps (i.e., with 2 sweeps per session excluded for each resampling average). The jitters of the resampled peak latencies followed a bell-shape distribution, with its width becoming larger as stimulus eccentricity increases (Figure 7A), because of larger inter-sweep variability. Hearing cats showed a smaller inter-sweep variability, especially at near-peripheral visual field (15- and 30-degree eccentricities) than deaf cats. This trend was even more apparent when the mean absolute deviations (MADs) of the jitters were quantified and plot as a function of the peak amplitude (Figure 7B). We performed an ANCOVA analysis regarding the effect of early deafness on MADs of latency jitter with peak amplitude as a covariate. We found a significant increase of MADs in early deaf cats compared to hearing cats for only P1 component [*F*(1,72) = 8.52, *p* = 0.005 < 0.01]. N1 component did not reveal such difference of statistical significance, neither was P1 component without holding peak amplitude as a covariate. Our findings revealed an increase in VEP variability for early deafness that is specific to the P1 component. The larger variation identified within each deaf cat subject was similar to the larger individual differences in the VEP topographical distribution found in cochlear implanted subjects (Buckley and Tobey, 2011).

## Discussion

In this study, we presented a checkerboard stimulus at seven different eccentricities, ranging from 0 to 90 degrees away from the midline, and compared the pattern-reversal visually evoked potentials (VEPs) between hearing and deaf cats. Overall, we observed no significant difference in response RMS values between the two groups, although some waveform components had larger amplitudes and shorter latencies in the deaf group, particularly for near- or mid-peripheral eccentricities. However, the inter-sweep variation analysis showed more variable latency in the deaf group for the peak component occurring about 100-ms post-stimulus.

### The effect of deafness on VEPs

Considering previous findings in both behavioral studies (Lomber et al., 2010) as well as human VEP studies (Neville et al., 1983; Hauthal et al., 2014), it might be expected that VEPs would be larger in the deaf compared to hearing cats, especially at far-peripheral (60-, 75-, 90-degree) eccentricities. However, as we proposed in the introduction and evidenced by our data, that may not be the case.

In the visual localization task of an earlier study (Lomber et al., 2010), animal subjects were alert and motivated by reward in orienting/approaching the locations of visual stimuli, where the visual stimuli had small size (less than 1-degree) and locations randomized trial to trial. In the VEP recording of the current study, animal subjects were sedated, not previously trained on a visual localization task, and the stimuli were larger in size (6 degree) and repeated for 20 trials at the same location.

When subjects are neither alert nor responsive, visual neurons with motor functions involved in eye movement tasks may make no or little contribution to the visual responses evoked by stimulus. While there is little information on how head movements modulate cortical visual neurons in cats (Straschill and Schick, 1977), many studies have shown the effect of eye movements on visual neuron in both cats (Guitton and Mandl, 1978a,b; Toyama et al., 1984; Yin and Greenwood, 1992; Weyand and Gafka, 1998) and non-human primates (Colby and Goldberg, 1999; Glimcher, 2001; Munoz, 2002).

In macaque monkeys, a large proportion of neurons in the frontal eye field (FEF) show no response to visual stimuli but reliable pre-saccadic activities for certain areas of the visual field, which are therefore deemed “movement” neurons (Glimcher, 2001). In contrast, most neurons in the lateral intraparietal area (LIP) demonstrate visual receptive field and their responses are modulated by stimulus saliency and task-relevance (Colby and Goldberg, 1999).

Compared to hearing cats, visual orienting behavior is improved in deaf cats, where accuracies of orienting to peripheral targets is increased (Lomber et al., 2010). This implies that neuronal function must be enhanced in visual representations and/or motor planning. The deactivation of deaf PAF eliminated the improved performance in the peripheral visual field (Lomber et al., 2010), which suggests that neurons in PAF acquire some functions in visual representations and/or motor planning. While there is still no evidence of visual responsiveness of PAF neurons in either hearing or deaf cats, cortical deactivation experiments show that PAF in hearing animals is involved in auditory localization (Malhotra et al., 2004; Malhotra and Lomber, 2007) and that PAF neurons are better tuned for sound location than A1 or AAF (Harrington et al., 2008). Therefore, neurons in PAF in hearing cats may already carry information of space required for the motor planning in visually guided orienting behavior, and may acquire functions in processing visual inputs following deafness.

Cat area 6 has been suggested as a homologue to primate FEF. Microstimulation in the banks and fundus of the perisylvian sulcus or the ventral bank of the cruciate sulcus in anesthetized cats produces saccadic eye movements, neck EMG activity (Guitton and Mandl, 1978a,b), and neuronal activity in superior colliculus (Meredith, 1999). However, only a small fraction of neurons in these areas demonstrated pre-saccadic activity in alert cats, but with a larger population being visually responsive (Weyand and Gafka, 1998). Although some neurons showed preference for certain eye movement direction, there is a lack of evidence for the behavioral relevance of these neurons as in primate FEF. However, area 6 may still serve as another potential target of cross-modal plasticity following deafness, by actively participating in the motor planning of eye and head movement.

While further investigation is still needed to support this hypothesis, we propose that a large proportion of functional cross-modal plasticity in cortex following deafness may reside in neural circuits associated with motor planning rather than those associated with visual processing.

It is also worth noting that neuromodulation systems, such as the dopaminergic (Anderson et al., 1994; Weil et al., 2010) and noradrenergic (McLean and Waterhouse, 1994; Treviño et al., 2019) systems, associated with reward and alertness, respectively, are actively involved in visually guided behaviors, as well as general task performance. Therefore, another possible explanation for the increased accuracy of peripheral visual localization in deaf cats may be found during the task training process, where reward are used to maintain subjects’ alertness and motivation to perform the task.

### The influence of dexmedetomidine

We did not attribute our negative results to the use of anesthetic agent, i.e., dexmedetomidine, because the anesthesia or sedation induced by dexmedetomidine is uniquely different from the other anesthetics, such as propofol and isoflurane, which directly interact with the GABAergic system (Hemmings et al., 2005) and may profoundly attenuate VEPs depending on their dosage (Sebel et al., 1986; Tanaka et al., 2020). One study has shown that the administration of dexmedetomidine does not affect VEPs in spine surgery patients undergoing general anesthesia with propofol (Rozet et al., 2015).

Although the VEPs recorded under dexmedetomidine cannot explain the enhanced performance in visual localization, our data is still the first report in cats on how early deafness changes VEPs that reflects stimulus-driven, bottom-up neural processing of visual inputs. Except when stimuli were positioned at central location or with 90-deg eccentricity, the medians of RMS and peak amplitude from the deaf group were almost unanimously larger than those from the hearing group. Although no main effect of deafness was revealed by ANOVA tests, a trend to increase in RMS and peak amplitude as well as decrease in peak latency seemed to appear at certain stimulus eccentricities with medium to large effect size worth being reported. Considering the low statistical power caused by small sample size used in the current study, a large effect size should have been expected to reveal statistical significance and avoid false negative. Similar cases were reported with a comparable sample size in previous histological investigations (Kok et al., 2014; Chabot et al., 2015; Wong et al., 2015; Meredith et al., 2016; Butler et al., 2018). Whereas a number of cat visual areas demonstrated increased or decreased number of afferents in several auditory areas in deaf cats when compared to hearing cats, only a few of such changes were statistically significant, such as the projections from posterolateral lateral suprasylvian area (PLLS) to the dorsal zone (DZ) of auditory cortex (Kok et al., 2014) and the projections from anterolateral lateral suprasylvian area (ALLS) to the anterior auditory fields (Wong et al., 2015).

Over all seven quantifications of VEPs and all seven stimulus eccentricities, the effect size values derived from the comparisons between the hearing and the deaf groups are mostly smaller than 1.5, and correspondingly led to a statistic power less than 59.8%. According to our calculation, at least 60% more than the current sample size in the deaf group is needed to achieve a statistic power higher than 80%, with the estimate of the current effect size assumed.

Regardless of the discussion above, we cannot completely exclude the possibility that the cross-modal plasticity shown in congenitally deaf cats is absent in early deaf cats or not sensitive to EEG recording technique. However, we consider these are the least likely, since several previous human ERP studies revealed different VEPs between hearing and deaf subjects (Neville et al., 1983; Hauthal et al., 2014; Campbell and Sharma, 2016; Corina et al., 2017).

We did find a statistically significant increase of latency variation in deaf subjects. The variability in neural activity in response to visual stimuli can be contributed by both neural and non-neural factors. Synaptic transmission is proposed to be a main source of neural variability due to its cascade nature of cellular and molecular process (Ribrault et al., 2011). Although we did not reveal a distinctive response amplitude in VEP from the deaf group, the reorganized auditory cortex may still participate in the early visual processing subtly, which involves synaptic transmission with less time precision. The increase we found in inter-sweep variability in deaf subjects, differentiating with respect to different VEP components, suggests a potential temporal specificity along the timeline of cortical sensory processing. Several neurological disorders (e.g., autism) have been associated with the increase in neural variability, which may share a similar underlying mechanism with this finding (Dinstein et al., 2015).

### The effect of stimulus eccentricity

In this study, we also examined the effect of stimulus eccentricity on VEPs. This has been explored in previous studies (Albus, 1975; Van Essen et al., 1984; Harvey and Dumoulin, 2011; Ziccardi et al., 2015), but not at a range of eccentricity covering the entire quarter-sphere. To investigate the peripheral-specificity of cross-modal plasticity after deafness using VEPs, it is critical to understand how VEPs are affected by stimulus eccentricity in normal hearing subjects.

The contralateral visual field in the cat is represented by multiple cortical visual areas. Extracellular recording shows that whereas all of the visual areas studied are responsive to stimuli presented near central vision, only areas 17, 19, PMLS, and 21b are responsive to stimuli present beyond 70-degree eccentricity along the horizontal meridian (Tusa et al., 1981). Except for area 17, the visual field represented in other cortical areas is more limited to the horizontal meridian at more peripheral positions (Tusa et al., 1981).

In this study, we quantified the effect of stimulus eccentricity on VEPs with an exponential model and found good fitness. This was in good consistent with a recent study in human subjects, where P1-N1 peak-to-peak amplitudes per squared-degree visual field as a function of stimulus eccentricities up to 20 degrees were exponentially modeled (Ziccardi et al., 2015). Our results suggest that the exponential model for the effect of stimulus eccentricity on VEPs can be generalized to the full range of quarter-sphere on the horizontal in non-primate species.

The attenuated VEP to stimulus present at peripheral field compared to central field that we observed is most likely due to cortical magnification, first observed with extracellular recording, where a larger proportion of visual cortical area were found representing a rather small central (foveal) visual field (Daniel and Whitteridge, 1961). Alternatively, it can also be argued that, because neurons in the peripheral visual field also have larger receptive field size in comparison to the central field (Albus, 1975; Van Essen et al., 1984), peripheral visual input can activate additional neurons as non-optimal stimulus as an advantage over central visual inputs. In virtue of such a trade-off, the product of cortical magnification factor (distance in cortex per degree in visual field) and receptive field size, also known as point-image size, was approximately constant for varying eccentricity (Harvey and Dumoulin, 2011). Our data showed that cortical activity measured using VEPs was not constant for varying eccentricity as point-image size.

One of the factors that interplays with cortical magnification in the effect of stimulus eccentricity is the respective participation of parvocellular and magnocellular pathways. Compared to the magnocellular pathway, the parvocellular pathway has a larger dynamic range for luminance contrast. It has been shown that VEPs to foveal stimuli (<2.5-degree eccentricity) demonstrate a broader range in response amplitude to various checkerboard contrasts when compared with peripheral stimuli (Laron et al., 2009), suggesting a maximized participation of the parvocellular pathway for the central visual field. Although its dominance decreases with eccentricity, the parvocellular pathway remains the major contributor in terms of the signal energy of VEPs as peripheral as 6.4 degrees (Baseler and Sutter, 1997). Given this majority position and high contrast stimuli being used, the results presented here reflect the unequal participation of both pathways, with the parvocellular pathway playing a greater part. However, it would be of interest for future studies to quantify the contribution of the parvocellular pathway for central versus peripheral visual field visual localization in deaf subjects.

It is also expected that there may be mutual benefits between future cat VEPs studies of this type and the development of source estimation algorithms for cat EEG signals. In humans, source estimation has been applied to VEPs with prior knowledge of generator locations derived from other brain imaging techniques such as PET (Woldorff et al., 1997) and fMRI (Hagler et al., 2009). Both human and cat cerebral cortex are gyrencephalic, which is a significant factor that compromises the performance of the source estimation algorithms using the concentric multi-sphere head models (Whittingstall et al., 2003). Although the smaller size of the cat head would raise the required spatial resolution of the scanners, such disadvantage is in the trade-off with an easier accessibility to invasive techniques such as extracellular recordings. In the current study, the error in VEP measurements regarding the lack of spatial resolution and unknown locations and orientation of VEP generators inevitably complicated the interpretation of our results. However, we speculate that the use of VEPs in assessing visual functions across visual fields in human and animal subjects is a promising approach.

In conclusion, our investigation of pattern reversal VEPs in response to peripherally present checkerboards in hearing and deaf cats demonstrated little functional plasticity in cortical activity driven by visual stimuli, suggesting that the enhanced visual localization previously reported in congenitally deaf cats is primarily due to plasticity in the neural circuit controlling orientation behavior instead of the lower-level visual system. In the future, we hope to use VEP techniques (1) to dissociate perception from orientation action, and (2) to adopt multi-channel recording and source localization.

## Data availability statement

The raw data supporting the conclusions of this article will be made available by the authors, without undue reservation.

## Ethics statement

The animal study was reviewed and approved by McGill University Animal Care Committee–DOWB.

## Author contributions

TM, XB, and SL contributed to the conception of research question and design of the experiment. TM and PB carried out data collection and participated in editing the manuscript. XB and TM performed the data analysis. XB and SL drafted the manuscript. All authors contributed to the article and approved the submitted version.
